# Symptom control in comprehensive conservative care for chronic kidney disease

**DOI:** 10.1590/2175-8239-JBN-2025-0260en

**Published:** 2026-11-06

**Authors:** Thaísa Leite Valverde, Patrícia Ferreira Abreu, Cibele Isaac Saad Rodrigues, Leda Lotaif, Christiane Ishikawa Ramos, Janaína Garcia Gonçalves, João Marcos da Costa Lucena, Camille Pereira Caetano, Renato Watanabe, Mila Paixão Sallenave, Eduardo Miranda, Tiago Magalhães, Lorena Porto Magalhães, Marina Aline Occhiena de Oliveira Neiva

**Affiliations:** 1 Sociedade Brasileira de Nefrologia, Comitê de Cuidados Paliativos, São Paulo, SP, Brazil.; 2 Universidade Federal de Sergipe, São Cristóvão, SE, Brazil.; 3 Universidade Federal de São Paulo, São Paulo, SP, Brazil.; 4 Pontifícia Universidade Católica de São Paulo, Faculdade de Ciências Médicas e da Saúde, Sorocaba, SP, Brazil.; 5 Hospital do Coração, São Paulo, SP, Brazil.; 6 Hospital Municipal Dr. Gilson de Cássia Marques de Carvalho, São Paulo, SP, Brazil.; 7 Universidade Federal do Rio Grande do Norte, Natal, RN, Brazil.; 8 Faculdade Nove de Julho Bauru, Bauru, SP, Brazil.; 9 Universidade Santo Amaro, São Paulo, SP, Brazil.; 10 Clínica Florence – Reabilitação e Cuidados Paliativos, Salvador, BA, Brazil.; 11 Centro Universitário Christus, Fortaleza, CE, Brazil.; 12 Hospital Universitário Prof. Edgar Santos, Salvador, BA, Brazil.; 13 Hospital Santa Casa de Misericórdia de Goiânia, Goiânia, GO, Brazil.

**Keywords:** Renal Insufficiency, Chronic, Palliative Care, Comprehensive Conservative Management

## Abstract

Over recent decades, the prevalence of chronic kidney disease (CKD) has risen markedly. In the terminal stage of the disease, renal replacement therapy (RRT) is traditionally recommended as the standard therapeutic approach. However, in older individuals with a high burden of comorbidities or substantial functional impairment, the benefits of RRT in terms of survival prolongation or improvement in quality of life become uncertain. In this context, comprehensive conservative kidney management (CKM) has emerged as a clinical alternative that is centered on the patient’s needs, values, and preferences. CKM entails an active, nondialytic management strategy for CKD that incorporates palliative care principles, with emphasis on kidney supportive care, slowing disease progression, and symptom control. This article discusses the main symptoms experienced by patients with advanced CKD managed conservatively and provides practical recommendations for their clinical management.

## INTRODUCTION

The global prevalence of chronic kidney disease (CKD) has increased significantly, mainly driven by population aging and the rising prevalence of comorbidities such as diabetes mellitus and hypertension^1^.

In the more advanced stages of CKD, kidney replacement therapy (KRT) constitutes a therapeutic option; however, not all patients are suitable candidates. Recent evidence has shown that older individuals with multiple comorbidities and/or significant functional impairment often derive limited benefits in terms of survival or quality of life from the initiation of KRT^2^. In this context, comprehensive conservative care (CCC) has emerged as a viable alternative for these patients, as well as for those who choose not to undergo dialysis treatment^3^.

The components of CCC include kidney supportive care (KSC), management of disease progression, and treatment of complications, all aligned with the patient’s individual goals and expectations, without the inclusion of KRT. KSC focuses on prioritizing quality of life, encompassing aspects such as shared decision-making, symptom management, crisis and advance care planning, spiritual support, integration with community services, and considerations related to end-of-life care and bereavement support^3,4,5^.

Appropriate symptom management is essential, as symptoms significantly impact both quality of life and functional status in these patients. The symptom burden and impairment in quality of life experienced by patients with CKD are comparable to those observed in patients with advanced cancer. Nevertheless, of particular concern is that approximately half of these symptoms remain inadequately treated^6^. Murtagh et al. reported that, during the last month of life, patients with CKD experienced an average of 20 symptoms, with pruritus, drowsiness, and dyspnea being the most prevalent^4^.

To improve the identification and monitoring of symptoms, disease-specific assessment tools have been developed, among which the Edmonton Symptom Assessment System Revised (ESAS-r) and the Integrated Palliative Care Outcome Scale Renal (IPOS-Renal) stand out. Both instruments have been translated, culturally adapted, and validated for use in Brazilian Portuguese^7^.

Therefore, early recognition and appropriate management of symptoms in patients with CKD receiving conservative care are essential.

In this review article, we discuss the most common symptoms encountered in advanced CKD and provide practical recommendations for their management, while acknowledging the limited availability of robust scientific evidence in certain clinical scenarios.

## FATIGUE

Fatigue is characterized by a subjective sensation of tiredness, weakness, or lack of energy that is disproportionate to the level of activity performed and not relieved by rest. It is a common symptom in advanced illnesses and one of the most disabling, with a substantial impact on functional status, autonomy, and quality of life^8^.

In a systematic review, fatigue was the most prevalent symptom (70%) among patients with CKD who were not receiving KRT^9^. Factors associated with the development and severity of fatigue in patients with CKD receiving CCC include a higher burden of comorbidities, antidepressant use, and lower hemoglobin levels. Furthermore, fatigue was independently associated with adverse outcomes, including CKD progression, increased mortality, and higher hospitalization rates^10^.

Nonpharmacological interventions are considered first-line therapy for the management of fatigue, although data specifically addressing CKD remain limited. In a randomized clinical trial, lower limb ergometric exercise performed during the first hour of hemodialysis sessions resulted in significant improvements in fatigue and functional performance after eight weeks^11^. Evidence from other populations with advanced illnesses suggests potential benefits from strategies such as regular physical activity, yoga, and psychotherapeutic interventions. Sleep hygiene measures, including limiting daytime naps and avoiding stimulants before bedtime, should also be encouraged^12^.

The main pharmacological approach consists of treating CKD-related complications, such as anemia, metabolic acidosis, and mineral and bone disorders, in addition to addressing associated conditions such as sleep disorders and depression. Among these interventions, treatment of anemia with erythropoiesis-stimulating agents appears to have the greatest impact on reducing fatigue. Although iron supplementation is an integral component of anemia management, consistent evidence supporting its direct effect on improvement in fatigue remains lacking^13^.

To date, there is no robust evidence supporting the routine use of pharmacological agents specifically for the treatment of fatigue in patients with CKD receiving CCC. Some medications have shown promising results in other clinical settings, such as amantadine in multiple sclerosis and methylphenidate in patients with cancer. Other agents, including dexamethasone, methylprednisolone, acetylsalicylic acid, L-carnitine, and modafinil, have also been investigated, with encouraging preliminary findings; however, CKD-specific studies are still needed before these therapies can be routinely recommended^14^.

## PRURITUS

Uremic pruritus is a common and potentially debilitating symptom that significantly affects quality of life. Data from the Chronic Kidney Disease Outcomes and Practice Patterns Study (CKDopps) indicate that 51% of patients with CKD report some degree of pruritus, which is classified as moderate to severe in 24% of cases. The study also demonstrated an association between pruritus and adverse outcomes, including poorer sleep quality and a higher prevalence of depressive symptoms. Patients with advanced CKD, of older age, and with multiple comorbidities are at increased risk of developing this symptom^15^.

The pathophysiology of uremic pruritus is complex and remains incompletely understood. Proposed mechanisms include disturbances in mineral and bone metabolism, accumulation of uremic toxins, peripheral neuropathy, immune dysregulation, and overstimulation of opioid receptors. Pruritus may occur in the absence of visible skin lesions; however, xerosis is present in 50% to 85% of cases and contributes substantially to symptom severity^16^.

Management should be individualized and patientcentered, beginning with the exclusion of other causes of pruritus, such as malignancies, hematologic disorders, liver disease, drug reactions, scabies, and allergies. General measures include avoiding triggering factors, such as extreme temperatures and excessive use of soaps, as well as implementing basic skin care, including regular moisturization with water-based emollients or topical treatment with 0.03% capsaicin, 1% hydrocortisone, or combinations of menthol, camphor, and phenol (0.3% each). Nonpharmacological interventions, such as ultraviolet B phototherapy and acupuncture, may also provide benefits. bone metabolism parameters is essential^3^.

First-line pharmacological treatment consists of gabapentin at doses ranging from 50 to 300 mg/day, preferably administered approximately two hours before bedtime. As an alternative, tricyclic antidepressants at doses of 10 to 25 mg/day may be used, also administered at night. Hydroxyzine may be considered as adjunctive therapy, and, in cases of opioid-induced pruritus, the addition of ondansetron is recommended^3^.

## PAIN

Pain is a subjective and multidimensional symptom resulting from the interaction of physical, psychological, social, and spiritual factors and is frequently associated with depression and anxiety. It is a common symptom among patients with CKD, with chronic pain affecting approximately 60% of patients and moderate-to-severe pain occurring in nearly 35% of cases^17^.

Pain assessment should encompass three main dimensions: intensity, type, and chronicity^18^. Pain intensity directly guides therapeutic decisions and should follow the World Health Organization analgesic ladder adapted for CKD. Numerical rating scales are widely used in clinical practice: scores ranging from 0 to 3 indicate mild pain, which generally does not require initiation or adjustment of therapy; scores ranging from 4 to 6 characterize moderate pain and indicate the need to initiate or modify treatment; and scores ranging from 7 to 10 correspond to severe pain requiring immediate intervention^18,19^.

Identification of the type of pain is equally important. Nociceptive pain tends to respond to nonopioid analgesics and opioids, whereas neuropathic pain is often refractory to these medications and requires specific pharmacological approaches. Assessment of chronicity is also essential. In acute pain, early identification and treatment of the underlying cause are crucial to prevent chronification, whereas in chronic pain, the emotional component plays a central role and should be incorporated into the therapeutic plan^18^.

Among nonpharmacological interventions, physical therapy, aerobic exercise, massage therapy, acupuncture, music therapy, and cognitive behavioral therapy (CBT) deserve particular attention. CBT has been associated with reductions in pain intensity, improvements in quality of life, and decreased opioid requirements, in addition to providing further benefits such as improvements in symptoms of depression and anxiety, sleep quality, and overall well-being^20,21^.

For the pharmacological management of pain (Table 1), mild nociceptive pain should be treated with nonopioid analgesics such as dipyrone (metamizole) and acetaminophen, as well as topical nonsteroidal anti-inflammatory drugs (NSAIDs). Oral NSAID use in patients with CKD should be avoided or undertaken with extreme caution because of the risk of worsening kidney function, gastrointestinal bleeding, and adverse cardiovascular events^22^. These agents inhibit cyclooxygenase enzymes and reduce prostaglandin synthesis, thereby contributing to acute kidney injury, hypertension, and hyperkalemia^23^.

**Table 1:** Analgesia in CKD^22,23^.

	Initial dose	Maximum dose
**Nociceptive pain**		
Dipyrone	500–1,000 mg, 8–12 h	3 g/day
Paracetamol	500 mg, 6–8 h	3 g/day
Tramadol	25–50 mg/day	200 mg/day
Oxycodone	5 mg/day	20 mg/day
Methadone	2.5 mg, 8–12 h	60–80 mg/day
Buprenorphine	5 mcg/h	20 mcg/h
**Neuropathic pain**		
Gabapentin	100 mg/day	300 mg/day
Pregabalin	25 mg/day	75 mg/day
Amitriptyline	25 mg/day	100 mg/day
Venlafaxine	37.5 mg/day	112.5 mg/day

For moderate nociceptive pain, tramadol or oxycodone is recommended. In cases of severe nociceptive pain, treatment options include transdermal buprenorphine with weekly patch replacement, sublingual buprenorphine, or methadone. Although opioid therapy is feasible and, in some cases, necessary, prescribing opioids to patients with CKD requires careful consideration because of the increased risk of adverse effects related to reduced excretion and accumulation of active metabolites^22,23^.

For neuropathic pain, therapeutic options include gabapentinoids such as gabapentin and pregabalin, tricyclic antidepressants such as amitriptyline, and serotoninnorepinephrine reuptake inhibitors such as venlafaxine^23^.

## DYSPNEA

Dyspnea is among the most prevalent and debilitating symptoms in patients with CKD, and its etiology is frequently multifactorial. Although volume overload and pulmonary edema are common causes, other conditions should also be considered, including anxiety, pain, panic attacks, anemia, infections, and metabolic acidosis^4,24^.

The management of dyspnea should involve the identification and, whenever possible, treatment of reversible causes. The therapeutic approach should integrate both nonpharmacological and pharmacological interventions. Recommended strategies include measures associated with subjective improvement in breathlessness as reported by patients, such as positioning with the head of the bed elevated; using handheld fans directed toward the face or remaining in well-ventilated environments; administering supplemental oxygen, preferably via nasal cannula; and using complementary interventions such as acupuncture, relaxation techniques, music therapy, and respiratory physiotherapy. Additional measures that may improve comfort include dietary sodium and fluid restriction, as well as avoidance of unnecessary intravenous fluid administration^4,24^.

Pharmacological management includes the use of loop diuretics when dyspnea is associated with hypervolemia and the patient has residual urine output. Benzodiazepines may be useful in alleviating the anxiety component, which is frequently present in this setting^4,25^. Near the end of life, low-dose opioids represent the most effective pharmacological approach for the control of dyspnea. However, their use in patients with CKD requires caution because of the risk of accumulation of active metabolites and toxicity. Fentanyl is generally considered the safest opioid in this context. In contrast, morphine and hydromorphone should be prescribed with careful dose adjustments, taking into account reduced renal clearance and the increased risk of adverse effects^4,24,25,26^.

In cases of refractory dyspnea, palliative sedation may be considered as an exceptional measure aimed at relieving the patient’s physical and psychological suffering by reducing the level of consciousness. This intervention should be undertaken with the consent of the patient and/or their legal representative and should involve consensus among the multidisciplinary team responsible for the patient’s care^27^.

## SLEEP DISORDERS

Sleep disorders are common among patients with CKD, including those in the early stages of the disease, with an estimated prevalence ranging from 40% to 80%. Despite this high prevalence, they frequently remain underdiagnosed and undertreated. The main sleep abnormalities include increased sleep latency, reduced sleep duration and efficiency, and frequent nocturnal awakenings, contributing to chronic fatigue, excessive daytime sleepiness, impaired quality of life, and increased mortality^28,29^.

The pathophysiology of sleep disorders in CKD is multifactorial and involves the accumulation of uremic toxins, electrolyte disturbances, chronic inflammation, neuroendocrine alterations, psychiatric comorbidities, and adverse drug effects. Sleep disorders are often associated with cardiovascular disease and psychosocial impairment.

The most common sleep disorders in CKD are insomnia, restless legs syndrome (RLS), and obstructive sleep apnea (OSA).

Insomnia is particularly prevalent in advanced CKD and among patients receiving dialysis. It is characterized by prolonged sleep latency, fragmented sleep, and daytime sleepiness. Associated factors include poor sleep habits, daytime napping, OSA, RLS, uremic pruritus, hypervolemia, dyspnea, pain, nocturia, mood disorders, and adverse medication effects^30^. Alterations in melatonin secretion, elevated orexin levels, and chronic inflammation appear to play a central role in the pathophysiology of insomnia^29^.

OSA has a bidirectional relationship with CKD: it contributes to disease progression through intermittent hypoxia and neurohormonal activation, while CKD-related volume overload promotes both OSA and central sleep apnea^31^. ts prevalence ranges from 34% to 65% in the early stages of CKD and reaches up to 56% among patients undergoing hemodialysis. OSA substantially increases cardiovascular morbidity and mortality and significantly impairs quality of life^32^.

Management begins with the identification of underlying causes and modifiable factors through a targeted clinical history, laboratory evaluation, and polysomnography when indicated^33^. Nonpharmacological treatment includes sleep hygiene measures, cognitive behavioral therapy (CBT), relaxation techniques, and regular physical activity^34^.

Pharmacological treatment should be individualized. Correction of anemia and iron deficiency improves symptoms of RLS and dyspnea, with a positive impact on sleep quality^35^. Associated conditions such as chronic pain, peripheral neuropathy, and uremic pruritus should be adequately treated, with low-dose gabapentin being a commonly used option^36^. Melatonin is safe and effective in reducing sleep latency and regulating the sleep-wake cycle^37^. Benzodiazepines and nonbenzodiazepine hypnotics (Z-drugs) should be used with caution because of the risk of excessive sedation and cognitive impairment, particularly in patients with CKD^38^. The treatment of OSA follows the recommendations for the general population and includes continuous positive airway pressure therapy and nocturnal oxygen supplementation when indicated^31^.

## RESTLESS LEGS SYNDROME

Restless legs syndrome (RLS) is characterized by an irresistible urge to move the lower limbs, frequently accompanied by unpleasant sensations that worsen during periods of rest, predominate in the evening or at night, and are relieved by movement. It affects 25% to 50% of patients with CKD receiving conservative care^39^.

The diagnosis is clinical and is based on medical history and the criteria established by the International Restless Legs Syndrome Study Group, requiring the presence of all five essential criteria (Chart 1)^40^. For screening purposes, a validated question with high sensitivity (100%) and specificity (96.8%) may be used: “When trying to relax or fall asleep, do you experience discomfort or restlessness in your legs that improves with movement?”^41^.

**Chart 1:** Essential diagnostic criteria for restless legs syndrome (all criteria must be met)^48^.

1. An urge to move the legs, usually, but not always, accompanied by or seemingly caused by uncomfortable and unpleasant sensations in the legs.
2. The urge to move the legs and any accompanying unpleasant sensations begin or worsen during periods of rest or inactivity, such as lying down or sitting.
3. The urge to move the legs and any accompanying unpleasant sensations are partially or totally relieved by movement, such as walking or stretching, at least for as long as the activity continues.
4. The urge to move the legs and any accompanying unpleasant sensations during rest or inactivity occur exclusively or predominantly in the evening or at night rather than during the day.
5. The occurrence of the above features is not solely accounted for by symptoms primary to another medical or behavioral condition (e.g., myalgia, venous stasis, leg edema, arthritis, leg cramps, positional discomfort, or habitual foot tapping).

RLS significantly impairs quality of life by disrupting sleep, increasing daytime fatigue, and negatively affecting mood, cognition, and daily functioning. Its pathophysiology in CKD is multifactorial, with central dopaminergic dysfunction, possibly related to cerebral iron deficiency, accumulation of uremic toxins, and fluid and electrolyte disturbances, particularly among patients undergoing dialysis, playing important roles^42^.

Differential diagnoses, including nocturnal leg cramps, painful peripheral neuropathy, myofascial pain syndromes, fibromyalgia, and akathisia, should be excluded^40^.

Treatment is indicated when symptoms significantly impair quality of life, sleep, or daytime functioning. Correction of iron deficiency should be prioritized, even in the absence of anemia, when serum ferritin levels are < 75 µg/L and/or transferrin saturation is < 20% to 25%. Potentially aggravating medications should be reviewed, including antipsychotics, antihistamines, metoclopramide, antidepressants (selective serotonin reuptake inhibitors, mirtazapine, and tricyclic antidepressants), carbamazepine, and lithium^43,44^.

Pharmacological options include dopamine agonists such as pramipexole (0.125–0.75 mg/day), ropinirole (0.25–3 mg/day), and rotigotine (1–3 mg/day). Although effective, prolonged use in patients with CKD requires caution because of the risk of augmentation, characterized by earlier onset of symptoms in the afternoon, worsening symptom severity, and possible involvement of the upper limbs^35,43,45^.

Gabapentin (up to 300 mg/day) and pregabalin (up to 75 mg/day) are effective and generally well-tolerated alternatives. Opioids should be reserved for refractory cases because of the risks of dependence and adverse events. Options include codeine (30–90 mg/day), tramadol (50–100 mg/day), and oxycodone (5–20 mg/day), with close monitoring recommended^35,43,45^.

Benzodiazepines and Z-drugs may be used intermittently, particularly when RLS is associated with insomnia or anxiety. Short-acting agents, such as zolpidem and zaleplon, are preferred and should be used cautiously in older adults because of the risk of falls, mental confusion, and complex sleep behaviors. Long-acting agents, such as clonazepam, should generally be avoided^43^.

Nonpharmacological measures include regular physical activity, sleep hygiene, and avoidance of stimulating substances. Massage therapy, pneumatic compression devices, and alternating heat and cold applications may provide additional symptomatic relief^35,45^.

In patients with advanced CKD, RLS may be profoundly disabling and often requires an individualized and multidisciplinary approach. Patient education, psychosocial support, and, in refractory cases, a focus on comfort and quality of life are fundamental components of care^46^.

## NAUSEA AND VOMITING

Nausea and vomiting are common symptoms among patients receiving CCC and individuals with CKD and are associated with substantial discomfort, impaired quality of life, and an increased risk of complications such as dehydration, electrolyte disturbances, and malnutrition^47^. The estimated prevalence of nausea and vomiting in this population is approximately 46% and 23%, respectively^46^.

Identification of the underlying etiology is essential for appropriate management. Symptoms may result from metabolic disturbances related to uremia, as well as associated conditions such as medication use, gastroparesis, and constipation^26,47^.

Medication-induced nausea and vomiting, particularly when associated with opioids, dopamine agonists, and certain antidepressants, are generally transient and typically last less than one week^26^. Opioids, which are widely used for pain management in advanced CKD, are associated with these symptoms in up to 60% of cases through stimulation of the chemoreceptor trigger zone (CTZ), increased vestibular sensitivity, and delayed gastric emptying.

Gastroparesis, characterized by early satiety and postprandial fullness, and constipation, through impaired gastrointestinal motility and bowel distension, also contribute substantially to these symptoms^47,48^.

Given the increased susceptibility of these patients to adverse effects, nonpharmacological interventions should be prioritized, either as standalone therapies or as adjuncts to pharmacological treatment^3,26^. These measures include appropriate oral hygiene, the use of sodium bicarbonate mouthwash, stimulation of salivation with chewing gum or mint, consumption of smaller and more frequent meals, slow food intake, and maintenance of an upright posture after meals. Alcohol, fatty, sweet, and spicy foods should be avoided, and exposure to strong odors should be minimized. Complementary therapies such as relaxation techniques, music therapy, hypnosis, and acupuncture may also be considered^3,26,33^.

Pharmacological treatment may be empirical or directed according to the most likely emetogenic pathway. Two major centers within the brainstem participate in the control of vomiting: the vomiting center, which integrates stimuli from the cerebral cortex, vestibular system, and gastrointestinal tract; and the CTZ, which is located outside the blood-brain barrier and is sensitive to toxins, medications, and biochemical disturbances^47^.

The concomitant use of antiemetics with similar receptor profiles is not recommended. In cases of persistent vomiting, impaired absorption, or significant gastric stasis, parenteral administration is preferred. Antiemetics should be prescribed on a regular schedule, titrated according to clinical response, and reassessed frequently for efficacy and adverse effects^47^.

Dopamine receptor antagonists, including domperidone, metoclopramide, and haloperidol, possess both antiemetic and prokinetic properties. Domperidone should be administered approximately 30 minutes before meals. Haloperidol is considered the treatment of choice for nausea and vomiting associated with uremia; however, it carries a higher risk of extrapyramidal adverse effects, particularly with prolonged use. Therefore, it should be avoided in patients with RLS or in those receiving dopamine agonists, and the concomitant use of these medications is not recommended. Olanzapine may represent an alternative with a lower risk of extrapyramidal effects, although it is associated with metabolic adverse effects^26,46^.

Antihistamines act primarily on the vestibular system and histamine H1 receptors and may be useful in selected situations; however, they are frequently associated with anticholinergic adverse effects and sedation^48^.

5-HT3 receptor antagonists, such as ondansetron, block both peripheral and central serotonergic pathways and may exacerbate constipation, while also increasing the risk of QT interval prolongation, particularly when combined with other medications with similar effects^3,47^.

## DEPRESSIVE AND ANXIETY SYNDROMES

Depressive and anxiety disorders are highly prevalent among patients receiving CCC and individuals with CKD, although they frequently remain underdiagnosed and undertreated. Studies have demonstrated a high burden of these disorders in CKD, with the prevalence of depressive symptoms ranging from 35% to 58% and that of anxiety symptoms ranging from 38% to 51%, particularly among patients receiving KRT^49,50,51^. Although data specifically addressing CCC in CKD are limited, it is plausible that the prevalences are comparable or even higher in advanced stages of the disease, particularly among patients managed conservatively^49,50,51^.

Depressive syndromes substantially impair quality of life, diminish the capacity to experience pleasure and derive meaning from the illness experience, interfere with the process of closure and life completion, and intensify the emotional burden experienced by family members and caregivers^52^.

The management of depressive and anxiety disorders in patients with CKD receiving CCC is challenging because of their association with worse clinical outcomes and increased suffering. Potentially reversible causes of anxiety and agitation should be investigated, including pain, pruritus, urinary retention, constipation, insomnia, irritability, and adverse drug effects, particularly those of corticosteroids^22^. Careful differential diagnosis is essential, as conditions such as panic disorder, hyperactive delirium, akathisia, and social anxiety disorder may mimic anxiety, whereas grief, sadness, hypoactive delirium, dementia, and metabolic disturbances may be mistaken for depression^53,54^.

Systematic screening for depression and anxiety is recommended in all patients with CKD receiving CCC. Diagnostic assessment should follow the criteria established in the *Diagnostic and Statistical Manual of Mental Disorders, Fifth Edition, Text Revision* (DSM-5-TR), while validated instruments such as the Patient Health Questionnaire-9 (PHQ-9) and the Generalized Anxiety Disorder-7 (GAD-7) may serve as adjunctive tools for screening, assessment of symptom severity, and monitoring of treatment response^55^.

The management of depressive and anxiety disorders should be multidimensional and multidisciplinary, with an emphasis on quality of life and preservation of dignity. Psychosocial interventions, particularly cognitive behavioral therapy (CBT), have consistently demonstrated reductions in depressive and anxiety symptoms as well as improvements in quality of life and are recommended because of their favorable safety profile^56,57^. Existential psychotherapies, including dignity therapy, logotherapy, and life review therapy, may be particularly beneficial in the management of anxiety by facilitating meaning-making and promoting the alignment of personal values and care priorities^58^.

There is currently no robust evidence supporting a specific class of antidepressants as first-line therapy for patients with CKD receiving CCC^59^. Treatment selection should therefore be individualized, taking into account comorbidities, adverse effect profiles, potential drug interactions, kidney function, and patient preferences. Selective serotonin reuptake inhibitors (SSRIs) may be used with caution because CKD-related alterations in pharmacokinetics may increase the risk of adverse outcomes, including bleeding, cardiovascular events, and fractures^59,60^. In addition to sertraline, commonly used agents include citalopram, escitalopram, fluoxetine, and paroxetine. Other therapeutic options include serotonin-norepinephrine reuptake inhibitors (venlafaxine, duloxetine, and mirtazapine), atypical antidepressants (bupropion and trazodone), and tricyclic antidepressants such as amitriptyline, all requiring careful monitoring^60^.

For the management of anxiety, short-and intermediateacting benzodiazepines, including lorazepam, clonazepam, and diazepam, may be used cautiously because of the risks of excessive sedation, delirium, and falls. Slow dose titration, reduced starting doses, and frequent reassessment are recommended (Chart 2)^22,61^.

**Chart 2:** Antidepressants and anxiolytics in chronic kidney disease.

Drugs	Dialyzability	Dose (mg/day)	Comments
**Antidepressants**			
Sertraline	No	Starting dose: 25; CKD dose range: 50–200.	First-line. Generally safe.
Fluoxetine	No	20–60	No dose adjustment required in CKD.
Amitriptyline	No	Initial: 10 at night and adjust up to 150.	Not considered first-line therapy. Tricyclic antidepressant. Use with caution. Limited use.
Escitalopram	No	10–20	No dose adjustment required in CKD.
Citalopram	No	Initial: 10 at night and adjust up to 40.	Risk of QT interval prolongation (ECG). Use with caution.
Nortriptyline	No	Initial: 10 at night and adjust up to 75–100.	Not considered first-line therapy. Tricyclic antidepressant. Use with caution. Limited use.
Trazodone	No	Initial: 25–50 and adjust up to 300. For insomnia: maximum 150.	Not considered first-line therapy. Use with caution. Atypical antidepressant.
Duloxetine	No	Start: ≤ 30 at night up to 120. No adjustment until GFR > 30 mL/min/1.73 m2.	Not considered first-line therapy. Use with caution. Not recommended in CKD stage 5 and dialysis patients.
Mirtazapine	No	Start: 15 at night. Up to CKD IV: 15–45. CKD V and dialysis: correct by 50%.	Not considered first-line therapy. Use with caution. Long half-life with delayed onset of adverse effects.
Venlafaxine	No	Start: 37.5 at night up to 112.5 in divided doses. Up to CKD IV: reduce dose by 25%–50%. CKD V and dialysis: correct by 50%.	Not considered first-line therapy. Use with caution. In HD patients, administer the dose after the dialysis session.
**Anxiety and Insomnia**			
Lorazepam	No	Anxiety: 2–3 PO or SL, up to 2 mg every 6 hours. Insomnia: start 1–2 PO or SL.	Generally safe. Lower doses may be sufficient.
Zolpidem	No	Start: 5 at night, potentially reaching up to 10.	Generally safe. Lower doses may be sufficient.
Zopiclone	No	Start: 3.75 at night, potentially reaching up to 7.5.	Generally safe. Lower doses may be sufficient.
Clonazepam	No	Start: 0.25–0.50 and maintain 0.50–2 every 6 hours.	Use with caution. Lower doses may be sufficient.
Diazepam	No	5–10	Use with caution and avoid if possible. Lower doses may be sufficient.
Bupropion	No	150–450	Use with caution. Lower doses and/or less frequent administration may be required.

Source: Adapted from the *Palliative Care Formulary*, 8th Edition^61^.

Complementary nonpharmacological strategies, including regular physical exercise and acupuncture, have demonstrated benefits in reducing depressive and anxiety symptoms in patients with CKD^62,63^. Relaxation techniques, meditation, music therapy, spirituality, religiosity, and resilience-promoting interventions may also contribute to improvements in psychological well-being and quality of life^64,65,66^.

Strengthening social support networks and promoting integrated multidisciplinary care involving nephrologists, psychiatrists, psychologists, and other healthcare professionals are essential for for a comprehensive, humanized, and safe approach, minimizing risks, polypharmacy, and drug interactions^49^.

## CONSTIPATION

Constipation is a common complaint among patients with CKD, although its exact prevalence remains uncertain because of the heterogeneity of diagnostic criteria used and the scarcity of robust studies. The Rome IV criteria are widely adopted and define constipation based on the presence of at least two of the six specific symptoms (Chart 3)^67,68^.

**Chart 3:** Rome IV criteria for constipation. Adapted from Lacy and Patel^68^.

Presence of 2 or more symptoms: Straining during > 25% of bowel movements; Lumpy or hard stools in > 25% of bowel movements; Sensation of incomplete evacuation in > 25% of bowel movements; Sensation of anorectal obstruction or blockage in > 25% of bowel movements; Use of manual maneuvers to facilitate bowel movements in > 25% of bowel movements; Fewer than 3 bowel movements per week.

In CKD, constipation is associated with impaired quality of life, increased healthcare costs, and clinically relevant complications, including accumulation of uremic toxins and hyperkalemia. Its etiology is multifactorial and involves physical inactivity, inadequate dietary fiber intake, chronic medication use, diabetic neuropathy, hypothyroidism, malignancies, and electrolyte disturbances, particularly hypercalcemia^67^.

Management should prioritize the identification and correction of reversible causes, with an initial focus on nonpharmacological interventions. First-line measures include encouraging regular physical activity, optimizing fluid and dietary intake, and providing behavioral guidance such as responding promptly to the urge to defecate and adopting an appropriate posture during defecation.

From a nutritional perspective, the consumption of whole grains, fruits, vegetables, and legumes promotes intestinal transit due to their fiber and probiotic compound content. However, these foods are also important sources of potassium and phosphorus, requiring individualized nutritional assessment in patients with CKD^69^.

Strategies aimed at increasing dietary fiber intake while minimizing the risk of hyperkalemia include selecting raw fruits and vegetables with lower potassium content and preboiling vegetables and legumes to reduce their potassium concentration. Beans, an important source of dietary fiber in the Brazilian diet, may be consumed when appropriately prepared to reduce potassium content. Furthermore, it is important to note that plant-derived phosphorus has lower bioavailability, thus posing a lower risk of hyperphosphatemia.

Fiber supplementation, including psyllium, guar gum, and wheat bran, as well as the use of olive oil as a stool-softening agent, may be considered, provided they are introduced gradually and accompanied by monitoring of clinical response^69^.

In the absence of an adequate response to nonpharmacological measures, pharmacological treatment with osmotic laxatives, stimulant laxatives, and prokinetic agents may be required. In refractory cases, evaluation for pelvic floor dysfunction is recommended, as these disorders may contribute to the persistence of chronic constipation^68,70^.

## SEXUAL DYSFUNCTION (SD)

SD is highly prevalent in patients with CKD and is associated with anxiety, depression, and impaired quality of life^9^. In men, the main manifestations include erectile dysfunction (ED), ejaculatory disorders, hypoactive sexual desire, and reduced libido, while in women, dyspareunia, decreased vaginal lubrication, reduced sexual desire, and anorgasmia predominate^71,72^.

It is estimated that ED affects approximately 71% of men with CKD, reaching 68–92% among patients on hemodialysis. Ejaculatory disorders and anorgasmia are reported by approximately 59% of men on RRT^73,74^. Among women, limited data suggest an SD prevalence of up to 84% in patients on RRT^75^.

SD in CKD is multifactorial, involving physical, hormonal, psychological, and social components. Fatigue, chronic pain, and a high comorbidity burden, including diabetes mellitus, hypertension, obesity, neuropathies, cardiovascular diseases, and peripheral vascular diseases, contribute significantly to its occurrence^73^.

Endocrine alterations, particularly dysfunction of the hypothalamic-pituitary-gonadal axis, become more pronounced with the progression of CKD^76^. Frequently used medications, such as beta-blockers, diuretics, sympatholytics, corticosteroids, angiotensin-converting enzyme inhibitors, and angiotensin II receptor blockers, can also aggravate SD^77^.

In women, hypoestrogenism is a central factor, associated with premature menopause, reduced genital blood flow, decreased vaginal lubrication, and dyspareunia^73,78^. The genitourinary syndrome of menopause is common and includes genital symptoms (atrophy, dryness, burning, and dyspareunia) and urinary symptoms (dysuria, urinary urgency, urinary incontinence, and recurrent infections)^79^.

ED in advanced CKD results from a combination of hormonal alterations, endothelial dysfunction, autonomic neuropathy, and uremic neuropathy, with impairment of the penile neurovascular response^71,73,80^. Hyperprolactinemia, reduced testosterone, and alterations in the hypothalamic-pituitary-gonadal axis are frequent findings^80^.

The assessment should be active and empathetic, as symptoms are rarely reported spontaneously. The *International Index of Erectile Function* (IIEF) is a validated reference instrument; the IIEF-5 is useful for screening and monitoring ED severity, although it does not identify its etiology (Chart 4)^81^.

**Chart 4:** International Index of Erectile Function (IIEF) 5^81^.

**In the last 4 weeks:**	
**Item 1**	**How often were you able to get an erection during sexual activity?**
Response	0. No sexual activity 1. Almost never or never 2. A few times (much less than half the time) 3. Sometimes (about half the time) 4. Most times (much more than half the time) 5. Almost always or always
**Item 2**	**When you had erections with sexual stimulation, how often were your erections hard enough for penetration?**
Response	0. No sexual activity 1. Almost never or never 2. A few times (much less than half the time) 3. Sometimes (about half the time) 4. Most times (much more than half the time) 5. Almost always or always
**Item 3**	**When you attempted sexual intercourse, how often were you able to penetrate your partner?**
Response	0. No sexual activity 1. Almost never or never 2. A few times (much less than half the time) 3. Sometimes (about half the time) 4. Most times (much more than half the time) 5. Almost always or always
**Item 4**	**During sexual intercourse, how often were you able to maintain your erection after you had penetrated your partner?**
Response	0. No sexual activity 1. Almost never or never 2. A few times (much less than half the time) 3. Sometimes (about half the time) 4. Most times (much more than half the time) 5. Almost always or always
**Item 5**	**During sexual intercourse, how difficult was it to maintain your erection until the completion of intercourse?**
Response	0. No sexual intercourse 1. Extremely difficult 2. Very difficult 3. Difficult 4. Slightly difficult 5. Not difficult

Management should be individualized and include psychosocial interventions and lifestyle changes. Optimized control of comorbidities and correction of anemia, mineral metabolism disorders, and hormonal alterations may result in improved sexual function. Smoking cessation, regular physical activity, and stress management are recommended.

For ED, the first-line treatment consists of phosphodiesterase type 5 inhibitors (PDE5i) (sildenafil, tadalafil, vardenafil, lodenafil), used on demand or, in the case of tadalafil, on a daily regimen. In patients with CKD, it is recommended to start with reduced doses, especially in those taking alpha-blockers or drugs that inhibit CYP3A4. The main contraindication is the concomitant use of nitrates^82^.

In the absence of a response, aspects related to the proper use of the medication should be reviewed, and hypogonadism should be evaluated, as its correction may improve therapeutic response. In refractory cases, intracavernous treatment or penile prosthesis implantation may be considered. Some patients show improvement after kidney transplantation.

Patients with moderate to severe refractory ED, confirmed hypogonadism, penile deformities, or suspected etiology unrelated to CKD should be referred to a specialist.

Management of dyspareunia includes vaginal lubricants and moisturizers and, for vaginal atrophy, topical estrogen, when there are no contraindications^83^. Pelvic floor physical therapy and CBT are effective strategies in the treatment of pain and associated psychosocial factors^83,84^.

## CONCLUSION

CKD is associated with a substantial symptom burden, particularly among older patients, those with multiple comorbidities, and those with functional impairment. In this context, comprehensive conservative care (CCC) represents a valid alternative to kidney replacement therapy (KRT). Systematic and proactive symptom assessment, combined with an individualized clinical approach, is essential for adequate symptom control and the delivery of person-centered care. CCC should be integrated early into the clinical trajectory, while respecting each patient’s values, preferences, and priorities.

## Data Availability

No new data were generated or analyzed in this study.
