# Supplementary material for: Symptom control in comprehensive conservative care for chronic kidney disease
Source: J Bras Nefrol. 2026 Nov 6;49(1):e20250260. doi: 10.1590/2175-8239-JBN-2025-0260en (PMC13743145; doi:10.1590/2175-8239-JBN-2025-0260en)
Supplement: Translation

# Translation: Controle de sintomas no manejo conservador abrangente na doença renal crônica

**DOI:** 10.1590/2175-8239-JBN-2025-0260pt

**Authors:** Thaísa Leite Valverde, Patrícia Ferreira Abreu, Cibele Isaac Saad Rodrigues, Leda Lotaif, Christiane Ishikawa Ramos, Janaína Garcia Gonçalves, João Marcos da Costa Lucena, Camille Pereira Caetano, Renato Watanabe, Mila Paixão Sallenave, Eduardo Miranda, Tiago Magalhães, Lorena Porto Magalhães, Marina Aline Occhiena de Oliveira Neiva

**Affiliations:** 1 Sociedade Brasileira de Nefrologia, Comitê de Cuidados Paliativos, São Paulo, SP, Brasil.; 2 Universidade Federal de Sergipe, São Cristóvão, SE, Brasil.; 3 Universidade Federal de São Paulo, São Paulo, SP, Brasil.; 4 Pontifícia Universidade Católica de São Paulo, Faculdade de Ciências Médicas e da Saúde, Sorocaba, SP, Brasil.; 5 Hospital do Coração, São Paulo, SP, Brasil.; 6 Hospital Municipal Dr. Gilson de Cássia Marques de Carvalho, São Paulo, SP, Brasil.; 7 Universidade Federal do Rio Grande do Norte, Natal, RN, Brasil.; 8 Faculdade Nove de Julho Bauru, Bauru, SP, Brasil.; 9 Universidade Santo Amaro, São Paulo, SP, Brasil.; 10 Clínica Florence – Reabilitação e Cuidados Paliativos, Salvador, BA, Brasil.; 11 Centro Universitário Christus, Fortaleza, CE, Brasil.; 12 Hospital Universitário Prof. Edgar Santos, Salvador, BA, Brasil.; 13 Hospital Santa Casa de Misericórdia de Goiânia, Goiânia, GO, Brasil.

**Keywords:** Insuficiência Renal Crônica, Cuidados Paliativos, Manejo Conservador Abrangente

## Abstract

Nas últimas décadas, a prevalência da doença renal crônica (DRC) tem aumentado de forma significativa. No estágio terminal da doença, a terapia renal substitutiva (TRS) é tradicionalmente indicada como abordagem terapêutica. Entretanto, em indivíduos idosos, com elevado número de comorbidades ou com limitação funcional significativa, os benefícios da TRS em termos de prolongamento da sobrevida ou de melhora da qualidade de vida tornam-se questionáveis. Nesse cenário, o manejo conservador abrangente (MCA) surge como uma alternativa clínica, centrada nas necessidades, valores e preferências do paciente. O MCA consiste em uma abordagem ativa da DRC sem o início da TRS, integrando os princípios dos cuidados paliativos, com foco no cuidado de suporte renal, na desaceleração da progressão da doença e no controle dos sintomas. Este artigo discute os principais sintomas observados em pacientes com DRC avançada sob manejo conservador e apresenta recomendações práticas para sua abordagem clínica.

## INTRODUÇÃO

A prevalência global da doença renal crônica (DRC) tem aumentado de forma significativa, impulsionada principalmente pelo envelhecimento populacional e pela maior incidência de comorbidades, como o diabetes mellitus e a hipertensão arterial^1^.

Nos estágios mais avançados da DRC, a terapia renal substitutiva (TRS) constitui uma opção terapêutica, embora nem todos os pacientes sejam elegíveis. Evidências recentes demonstram que indivíduos idosos, com múltiplas comorbidades e/ou comprometimento funcional significativo, frequentemente não apresentam ganhos relevantes em sobrevida ou qualidade de vida ao iniciar a TRS^2^. Assim, o manejo conservador abrangente (MCA) surge como uma alternativa viável para esses pacientes, bem como para aqueles que optam por não realizar o tratamento dialítico^3^.

Os componentes do MCA incluem cuidados de suporte renal (CSR), manejo da progressão da doença e controle de complicações, sempre alinhados às expectativas individuais do paciente, sem a inclusão da TRS. Os CSR têm como foco a priorização da qualidade de vida e abrangem aspectos como tomada de decisão compartilhada, manejo dos sintomas, planejamento para situações de crise e cuidados avançados, suporte espiritual, integração com serviços comunitários e considerações relacionadas aos cuidados de fim de vida e ao luto^3,4,5^.

O manejo adequado dos sintomas é essencial, uma vez que eles impactam significativamente a qualidade de vida e o *status* funcional desses pacientes. A carga sintomática e o comprometimento da qualidade de vida nos pacientes com DRC são compatíveis com os observados em pacientes com neoplasia avançada. Entretanto, de forma preocupante, cerca da metade desses sintomas permanece inadequadamente tratada^6^. Murtagh etal. identificaram que, no último mês de vida, pacientes com DRC apresentavam, em média, 20 sintomas, sendo prurido, sonolência e dispneia os mais frequentes^4^. Para aprimorar a identificação e o monitoramento desses sintomas, foram desenvolvidas escalas específicas, destacando-se o *Edmonton Symptom Assessment System (ESAS-r)* e a *Integrated Palliative Care Outcome Scale Renal (IPOS-Renal)*, ambas validadas para o português do Brasil^7^.

Dessa forma, a identificação precoce e o manejo adequado dos sintomas em pacientes com DRC em tratamento conservador são essenciais.

Neste artigo de revisão, abordaremos os sintomas mais frequentes nos estágios avançados da DRC e apresentaremos sugestões práticas para seu controle, considerando a limitação de evidências científicas robustas em algumas situações clínicas.

## FADIGA

A fadiga é caracterizada por uma sensação subjetiva de cansaço, fraqueza ou falta de energia, desproporcional à atividade realizada e não aliviada com o repouso. Trata-se de um sintoma frequente em doenças avançadas e um dos mais incapacitantes, com impacto significativo na funcionalidade, na autonomia e na qualidade de vida^8^.

Em revisão sistemática, a fadiga foi o sintoma mais prevalente (70%) em pacientes com DRC não submetidos à TRS^9^. Entre os fatores associados ao desenvolvimento e à intensidade da fadiga em pacientes com DRC em MCA, destacam-se o maior número de comorbidades, o uso de antidepressivos e os níveis reduzidos de hemoglobina. Além disso, a fadiga mostrou associação independente com desfechos adversos, incluindo progressão da DRC, aumento da mortalidade e maior taxa de hospitalização^10^.

As intervenções não medicamentosas são reconhecidas como de primeira linha no manejo da fadiga, embora existam dados limitados especificamente na DRC. Em ensaio clínico randomizado, exercícios físicos ergométricos de membros inferiores, durante a primeira hora da sessão de hemodiálise, resultaram em melhora significativa da fadiga e do desempenho funcional após oito semanas^11^. Evidências provenientes de outras populações com doenças avançadas sugerem benefícios de estratégias como atividade física regular, ioga e intervenções psicoterapêuticas. Medidas de higiene do sono, incluindo a restrição de cochilos diurnos e a evitação de estimulantes antes de dormir, também devem ser incentivadas^12^.

A principal abordagem farmacológica consiste no tratamento das complicações da DRC, como anemia, acidose metabólica e distúrbios mineral-ósseos, além do manejo de condições associadas, como distúrbios do sono e depressão. Entre essas intervenções, o tratamento da anemia com agentes estimulantes da eritropoiese apresenta o maior impacto na redução da fadiga. Embora a reposição de ferro integre o manejo da anemia, ainda faltam evidências consistentes sobre seu efeito direto na melhora desse sintoma^13^.

Não existem, até o momento, evidências robustas que sustentem o uso rotineiro de medicamentos para o tratamento da fadiga em pacientes com DRC em MCA. Algumas medicações demonstraram resultados promissores em outros contextos clínicos, como a amantadina na esclerose múltipla e o metilfenidato em pacientes oncológicos. Outros fármacos, incluindo a dexametasona, metilprednisolona, ácido acetilsalicílico, L-carnitina e modafinila, vêm sendo investigados, com achados iniciais favoráveis, embora ainda sejam necessários estudos específicos na DRC^14^.

## PRURIDO

O prurido urêmico é um sintoma frequente e potencialmente debilitante, com impacto significativo na qualidade de vida. Dados do estudo *Chronic Kidney Disease Outcomes and Practice Patterns Study* (CKDopps) indicam que 51% dos pacientes com DRC relatam algum grau de prurido, classificado como moderado a intenso em 24% dos casos.

O estudo também demonstrou associação do prurido com desfechos adversos, como pior qualidade do sono e maior prevalência de sintomas depressivos. Pacientes nos estágios avançados da DRC, mais idosos e com múltiplas comorbidades apresentam maior risco de desenvolver esse sintoma^15^.

A fisiopatologia do prurido urêmico é complexa e ainda não completamente compreendida. Os mecanismos envolvidos incluem alterações no metabolismo mineral e ósseo, acúmulo de toxinas urêmicas, neuropatia periférica, disfunções imunológicas e hiperestimulação dos receptores opioides. O prurido pode ocorrer na ausência de lesões cutâneas evidentes; contudo, a xerose está presente em 50% a 85% dos casos, intensificando o sintoma^16^.

A abordagem deve ser individualizada e centrada no paciente, iniciando-se pela exclusão de outras causas de prurido, como neoplasias, doenças hematológicas, hepatopatias, reações medicamentosas, escabiose e alergias. Medidas gerais incluem evitar fatores desencadeantes, como temperaturas extremas e uso excessivo de sabonetes, além de instituir cuidados básicos com a pele, com hidratação regular por meio de emolientes à base de água ou uso tópico de capsaicina a 0,03%, hidrocortisona a 1% ou combinações de mentol, cânfora e fenol (0,3% cada). Intervenções não farmacológicas, como fototerapia com ultravioleta B e acupuntura, podem apresentar benefícios. Adicionalmente, é fundamental o controle adequado dos parâmetros do metabolismo mineral-ósseo^3^.

O tratamento farmacológico de primeira linha consiste no uso de gabapentina, na dose de 50 a 300 mg/dia, preferencialmente administrada cerca de duas horas antes de dormir. Como alternativa, podem ser utilizados antidepressivos tricíclicos, em doses de 10 a 25 mg/dia, também à noite. A hidroxizina pode ser utilizada como adjuvante e, nos casos de prurido associado ao uso de opioides, recomenda-se a associação de ondansetrona^3^.

## DOR

A dor é um sintoma subjetivo e multidimensional, resultante da interação de fatores físicos, psicológicos, sociais e espirituais, frequentemente associada a quadros de depressão e ansiedade. Trata-se de um sintoma comum em pacientes com DRC, com ocorrência crônica em cerca de 60% deles, sendo moderada a grave em aproximadamente 35% dos casos^17^.

Sua avaliação deve abranger três dimensões principais: a intensidade, o tipo e a cronicidade^18^. A intensidade orienta diretamente a conduta terapêutica e deve seguir a Escala de Analgesia da Organização Mundial da Saúde adaptada para DRC. As escalas numéricas são amplamente utilizadas na prática clínica: escores de 0 a 3 indicam dor leve, geralmente sem necessidade de início ou ajuste terapêutico; valores de 4 a 6 caracterizam dor moderada, indicando a necessidade de iniciar ou modificar o tratamento; e escores de 7 a 10 correspondem à dor intensa que requer intervenção imediata^18,19^.

A identificação do tipo de dor é igualmente fundamental. A dor nociceptiva tende a responder a analgésicos não opioides e opioides, enquanto a dor neuropática é frequentemente refratária a esses medicamentos, demandando abordagens farmacológicas específicas. A avaliação da cronicidade também é essencial: na dor aguda, a identificação e o tratamento precoce da causa subjacente são fundamentais para prevenir a cronificação; já na dor crônica, o componente emocional assume papel central e deve ser integrado ao plano terapêutico^18^.

Entre as medidas não farmacológicas, destacam-se a fisioterapia, os exercícios aeróbicos, a massagem, a acupuntura, a musicoterapia e a terapia cognitivo-comportamental (TCC). A TCC está associada à redução da intensidade da dor, à melhora da qualidade de vida e à menor necessidade de opioides, além de benefícios adicionais como melhora dos sintomas de depressão e ansiedade, da qualidade do sono e do bem-estar geral^20,21^.

No tratamento farmacológico (Tabela 1) da dor nociceptiva leve, recomendam-se analgésicos não opioides, como dipirona e paracetamol, além de anti-inflamatórios não esteroidais (AINEs) tópicos. O uso oral de AINEs em pacientes com DRC deve ser evitado ou realizado com extrema cautela, devido ao risco de piora da função renal, sangramento gastrointestinal e eventos cardiovasculares adversos^22^. Esses fármacos inibem a enzima ciclo-oxigenase, reduzindo a síntese de prostaglandinas, o que pode contribuir para lesão renal aguda, hipertensão arterial e hipercalemia^23^.

**Tabela 1:** Analgesia na DRC^22,23^.

	Dose inicial	Dose máxima
**Dor nociceptiva**		
Dipirona	500–1.000 mg, 8–12 h	3 g/dia
Paracetamol	500 mg, 6–8 h	3 g/dia
Tramadol	25–50 mg/dia	200 mg/dia
Oxicodona	5 mg/dia	20 mg/dia
Metadona	2,5 mg, 8–12 h	60–80 mg/dia
Buprenorfina	5 mcg/h	20 mcg/h
**Dor neuropática**		
Gabapentina	100 mg/dia	300 mg/dia
Pregabalina	25 mg/dia	75 mg/dia
Amitriptilina	25 mg/dia	100 mg/dia
Venlafaxina	37,5 mg/dia	112,5 mg/dia

Nos casos de dor nociceptiva moderada, indica-se o uso de tramadol ou oxicodona. Para dor nociceptiva intensa, podem ser utilizados buprenorfina transdérmica, com troca do adesivo semanal, buprenorfina por via sublingual ou metadona. Embora o uso de opioides seja possível e, em alguns casos, necessário, sua prescrição em pacientes com DRC deve ser criteriosa, em razão do maior risco de efeitos adversos relacionados à redução da excreção e ao acúmulo de metabólitos ativos^22,23^.

Na dor neuropática, as opções terapêuticas incluem gabapentinoides, como gabapentina e pregabalina, antidepressivos tricíclicos, como a amitriptilina, e inibidores da recaptação de serotonina e noradrenalina, como a venlafaxina^23^.

## DISPNEIA

A dispneia destaca-se entre os sintomas mais prevalentes e debilitantes em pacientes com DRC, sendo sua etiologia multifatorial. Embora a sobrecarga volêmica e o edema pulmonar sejam causas frequentes, outras condições devem ser consideradas, como ansiedade, dor, crises de pânico, anemia, infecções e acidose metabólica^4,24^.

O manejo da dispneia deve envolver a identificação e, sempre que possível, o tratamento das causas reversíveis. A abordagem terapêutica deve integrar medidas não medicamentosas às medicamentosas. Dentre as estratégias recomendadas, destacam-se medidas associadas à melhora subjetiva da sensação de dispneia relatada pelos pacientes, como o posicionamento com cabeceira elevada; utilização de ventiladores portáteis direcionados ao rosto ou permanência em ambientes com melhor ventilação natural; a administração de oxigênio suplementar, preferencialmente por cateter nasal; além de intervenções complementares, como acupuntura, técnicas de relaxamento, musicoterapia e fisioterapia respiratória. Também podem contribuir para maior conforto a restrição de sódio e líquidos na dieta, bem como a não realização de infusões intravenosas desnecessárias^4,24^.

No manejo medicamentoso, indicam-se diuréticos de alça quando a dispneia está associada à hipervolemia e o paciente apresenta diurese residual. Benzodiazepínicos podem ser úteis no alívio do componente ansioso, frequentemente presente nesse contexto^4,25^. Próximo ao fim da vida, opioides em baixas doses constituem a abordagem medicamentosa mais eficaz para o controle da dispneia. No entanto, seu uso em pacientes com DRC requer precaução devido ao risco de acúmulo de metabólitos ativos e toxicidade. O fentanil é geralmente considerado o opioide mais seguro nesse cenário. Por outro lado, morfina e hidromorfona devem ser utilizadas com ajustes de dose criteriosos, considerando a depuração renal reduzida e o risco de efeitos adversos^4,24,25,26^.

Em casos de dispneia refratária, pode-se considerar a sedação paliativa como medida excepcional, com o objetivo de aliviar o sofrimento psíquico e físico do paciente por meio da redução do nível de consciência. Tal intervenção deve ser realizada com o consentimento do paciente e/ou de seu representante legal e deve contar com o consenso da equipe multiprofissional envolvida no cuidado^27^.

## DISTÚRBIOS DO SONO (DS)

Os DS são frequentes em pacientes com DRC, inclusive nos estágios iniciais, com prevalência estimada entre 40% e 80%.

Apesar disso, permanecem frequentemente subdiagnosticados e subtratados. As principais alterações incluem aumento da latência para início do sono, redução da duração e da eficiência do sono e despertares noturnos frequentes, contribuindo para a fadiga crônica, sonolência diurna excessiva, piora da qualidade de vida e aumento da mortalidade^28,29^.

A fisiopatologia é multifatorial, envolvendo acúmulo de toxinas urêmicas, distúrbios eletrolíticos, inflamação crônica, alterações neuroendócrinas, comorbidades psiquiátricas e efeitos adversos de medicamentos. Os DS estão frequentemente associados a doenças cardiovasculares e comprometimento psicossocial.

Os DS mais frequentes na DRC são insônia, síndrome das pernas inquietas (SPI) e apneia obstrutiva do sono (AOS).

A insônia é particularmente prevalente nos estágios avançados da DRC e entre pacientes em diálise. Caracteriza-se por latência aumentada, sono fragmentado e sonolência diurna. Seus fatores associados incluem maus hábitos de sono, cochilos diurnos, AOS, SPI, prurido urêmico, hipervolemia, dispneia, dor, nictúria, transtorno do humor e efeitos adversos de medicamentos^30^. Alterações na secreção de melatonina, níveis elevados de orexina e inflamação crônica parecem desempenhar papel fundamental na fisiopatologia da insônia^29^.

A AOS apresenta relação bidirecional com a DRC: contribui para sua progressão por meio da hipóxia intermitente e da ativação neuro-hormonal, enquanto a hipervolemia da DRC favorece tanto a AOS quanto a apneia central^31^. Sua prevalência varia entre 34% e 65% nos estágios iniciais da DRC, alcançando até 56% em pacientes em hemodiálise. A AOS agrava a morbimortalidade cardiovascular e compromete significativamente a qualidade de vida^32^.

O manejo inicia-se pela identificação de causas subjacentes e fatores modificáveis, por meio de anamnese dirigida, avaliação laboratorial e polissonografia quando indicada^33^. O tratamento não farmacológico inclui medidas de higiene do sono, TCC, técnicas de relaxamento e atividade física regular^34^.

A abordagem medicamentosa deve ser individualizada. A correção da anemia e da deficiência de ferro melhora os sintomas da SPI e da dispneia, com impacto positivo na qualidade do sono^35^. Condições associadas, como dor crônica, neuropatia periférica e prurido urêmico, devem ser tratadas adequadamente, sendo a gabapentina em doses baixas uma opção frequente^36^. A melatonina é segura e eficaz na redução da latência do sono e na regulação do ciclo sono-vigília^37^. Benzodiazepínicos e fármacos Z devem ser utilizados com cautela devido ao risco de sedação excessiva e comprometimento cognitivo, especialmente em pacientes com DRC^38^. Para a AOS, o tratamento segue as recomendações da população geral, incluindo pressão positiva contínua nas vias aéreas e oxigenoterapia noturna quando indicada^31^.

## SÍNDROME DAS PERNAS INQUIETAS (SPI)

A SPI é caracterizada por uma necessidade irresistível de mover os membros inferiores, frequentemente acompanhada por sensações desagradáveis, com piora em repouso, predominância noturna e alívio com o movimento. Acomete entre 25% e 50% dos pacientes com DRC em tratamento conservador^39^.

O diagnóstico é clínico, baseado na anamnese e nos critérios do *International Restless Legs Syndrome Study Group*, sendo obrigatória a presença dos cinco critérios essenciais (Quadro 1)^40^. Para triagem, pode-se empregar uma pergunta validada com alta sensibilidade (100%) e especificidade (96,8%): “Ao tentar relaxar ou dormir, você sente desconforto ou inquietação nas pernas que melhora ao se movimentar?”^41^.

**Quadro 1:** Critérios diagnósticos essenciais para SPI (todos devem ser atendidos)^48^.

1. Uma vontade de mover as pernas, geralmente, mas nem sempre, acompanhada ou aparentemente causada por sensações desconfortáveis e desagradáveis nas pernas.
2. A vontade de mover as pernas e quaisquer sensações desagradáveis que as acompanham começam ou pioram durante períodos de repouso ou inatividade, como ao deitar-se ou sentar-se.
3.A vontade de mover as pernas e quaisquer sensações desagradáveis que as acompanham são parciais ou totalmente aliviadas pelo movimento, como caminhar ou se alongar, pelo menos enquanto a atividade continuar.
4. A vontade de mover as pernas e quaisquer sensações desagradáveis que as acompanham durante o repouso ou a inatividade ocorrem ou são piores apenas no final do dia ou à noite, e não durante o dia.
5. A ocorrência das características acima não é atribuída a sintomas primários de outra condição médica ou comportamental (por exemplo, mialgia, estase venosa, edema nas pernas, artrite, cãibras nas pernas, desconforto posicional, hábito de bater os pés).

A SPI compromete significativamente a qualidade de vida, interferindo no sono, aumentando a fadiga diurna e impactando negativamente o humor, a cognição e a funcionalidade diária. Sua fisiopatologia na DRC é multifatorial, destacando-se a disfunção dopaminérgica central possivelmente relacionada à deficiência de ferro cerebral, ao acúmulo de toxinas urêmicas e aos distúrbios hidroeletrolíticos, especialmente em pacientes em diálise^42^.

Devem ser excluídos diagnósticos diferenciais, como cãibras noturnas, neuropatia periférica dolorosa, síndromes miofasciais, fibromialgia e acatisia motora^40^.

O tratamento é indicado quando há impacto significativo na qualidade de vida, no sono ou no funcionamento diurno. A correção da deficiência de ferro deve ser priorizada, mesmo na ausência de anemia, quando a ferritina estiver < 75 µg/L e/ou a saturação de transferrina < 20 a 25%. Medicamentos potencialmente agravantes devem ser revistos, incluindo antipsicóticos, anti-histamínicos, metoclopramida, antidepressivos (ISRS, mirtazapina, tricíclicos), carbamazepina e lítio^43,44^.

As opções farmacológicas incluem agonistas dopaminérgicos, como pramipexol (0,125–0,75 mg/dia), ropinirol (0,25– 3 mg/dia) ou rotigotina (1–3 mg/dia). Embora eficazes, seu uso prolongado requer cautela na DRC devido ao risco de “síndrome de aumento”, caracterizada pela antecipação dos sintomas para o período da tarde, intensificação das queixas e possível acometimento de membros superiores^35,43,45^.

Gabapentina (até 300 mg/dia) e pregabalina (até 75 mg/dia) são alternativas eficazes e bem toleradas. Opioides devem ser reservados para casos refratários, devido ao risco de dependência e de eventos adversos, podendo-se utilizar codeína (30–90 mg/dia), tramadol (50–100 mg/dia) ou oxicodona (5–20 mg/dia), com monitorização rigorosa^35,43,45^.

Benzodiazepínicos e fármacos Z podem ser utilizados de forma intermitente, sobretudo quando a SPI está associada à insônia ou à ansiedade. Opções de curta duração são preferíveis (zolpidem e zaleplon), com cautela em idosos, devido ao risco de quedas, confusão mental e comportamentos complexos do sono. Agentes de meia-vida prolongada, como clonazepam, devem ser evitados^43^.

Entre as medidas não medicamentosas, destacam-se a atividade física regular e a higiene do sono, além da recomendação de evitar o uso de substâncias estimulantes. Massagem, compressão pneumática e aplicação alternada de calor e frio podem oferecer alívio adicional^35,45^.

Em pacientes com DRC avançada, a SPI pode ser altamente incapacitante, exigindo uma abordagem individualizada e multidisciplinar. A educação do paciente, o suporte psicossocial e, nos casos refratários, o foco no conforto e na qualidade de vida são fundamentais^46^.

## NÁUSEA E VÔMITO

Náusea e vômito são sintomas frequentes em pacientes sob MCA e em indivíduos com DRC, associando-se a importante desconforto, piora da qualidade de vida e risco de complicações, como desidratação, distúrbios hidroeletrolíticos e desnutrição^47^. Estima-se prevalência de aproximadamente 46% para náusea e 23% para vômito nessa população^46^.

A identificação da etiologia é fundamental para o manejo adequado. Os sintomas podem decorrer de distúrbios metabólicos relacionados à uremia, além de causas associadas, como uso de medicamentos, gastroparesia e constipação^26,47^.

Náuseas e vômitos induzidos por medicamentos, principalmente opioides, agonistas dopaminérgicos e alguns antidepressivos, são geralmente transitórios, com duração inferior a uma semana^26^. Os opioides, amplamente utilizados para o controle da dor na DRC avançada, estão associados a esses sintomas em até 60% dos casos, por estimulação da zona de gatilho quimiorreceptora (ZGQ), aumento da sensibilidade vestibular e retardo do esvaziamento gástrico.

A gastroparesia, caracterizada por saciedade precoce e empachamento pós-prandial, e a constipação, por meio da motilidade gastrointestinal prejudicada e da distensão intestinal, também contribuem de forma relevante para esses sintomas^47,48^.

Diante da maior susceptibilidade a efeitos adversos, recomenda-se priorizar medidas não medicamentosas, isoladamente ou como complemento ao tratamento medicamentoso^3,26^. Incluem-se higiene oral adequada, uso de enxaguante bucal com bicarbonato de sódio, estímulo à salivação (chicletes ou hortelã), refeições fracionadas em porções menores, ingestão lenta dos alimentos e manutenção de postura ereta após as refeições. Deve-se evitar o álcool, alimentos gordurosos, doces ou apimentados e reduzir a exposição a odores fortes. Terapias complementares, como técnicas de relaxamento, musicoterapia, hipnose e acupuntura, podem ser consideradas^3,26,33^.

O tratamento medicamentoso pode ser empírico ou direcionado conforme a provável via emetogênica envolvida. Existem dois centros principais do tronco cerebral que participam do controle do vômito: o centro de vômito, que integra estímulos do córtex cerebral, do sistema vestibular e do trato gastrointestinal; e a ZGQ, localizada fora da barreira hematoencefálica e sensível a toxinas, drogas e distúrbios bioquímicos^47^.

Não é recomendado o uso concomitante de antieméticos com perfis de receptores semelhantes. Em casos de vômitos persistentes, má absorção ou estase gástrica importante, a via parenteral é preferível. Os medicamentos devem ser prescritos regularmente, titulados de acordo com a resposta e revisados frequentemente quanto à eficácia e aos efeitos adversos^47^.

Os antagonistas dopaminérgicos (domperidona, metoclopramida e haloperidol) apresentam efeito antiemético e procinético. A domperidona deve ser administrada 30 minutos antes das refeições. O haloperidol é o fármaco de escolha para náuseas e vômitos associados à uremia, porém apresenta maior risco de efeitos extrapiramidais, especialmente com o uso prolongado. Por isso, deve ser evitado em pacientes com SPI ou que estejam em uso de agonistas dopaminérgicos, não sendo indicada a associação dessas medicações. A olanzapina pode ser uma alternativa com menor risco de efeitos extrapiramidais, embora associada a efeitos adversos metabólicos^26,46^.

Os anti-histamínicos atuam no sistema vestibular e nos receptores de histamina H1, sendo úteis em situações selecionadas, mas frequentemente associados a efeitos anticolinérgicos e sedação^48^.

Os antagonistas dos receptores 5-HT3 (ex.: ondansetrona) bloqueiam vias serotoninérgicas periféricas e centrais e podem exacerbar a constipação, além de aumentar o risco de prolongamento do intervalo QT, especialmente quando associados a outros fármacos com o mesmo efeito^3,47^.

## SÍNDROMES DEPRESSIVAS E ANSIOSAS

Os transtornos depressivos e ansiosos são prevalentes em pacientes sob MCA e em indivíduos com DRC, embora frequentemente permaneçam subdiagnosticados e subtratados. Estudos demonstraram elevada ocorrência desses transtornos na DRC, com prevalência de sintomas depressivos variando entre 35% e 58% e a de ansiedade, entre 38% e 51%, especialmente entre pacientes em TRS^49,50,51^. Embora existam poucos dados específicos sobre MCA no contexto da DRC, é plausível que as prevalências sejam iguais ou superiores nos estágios avançados da doença, particularmente em pacientes em tratamento conservador^49,50,51^.

A síndrome depressiva compromete significativamente a qualidade de vida, reduz a capacidade de experimentar prazer e de atribuir significado à experiência de adoecimento, interfere no processo de despedida e intensifica o sofrimento dos familiares e cuidadores^52^.

O manejo desses transtornos em pacientes com DRC em MCA é desafiador, dada sua associação com piores desfechos clínicos e maior sofrimento. Devem ser investigadas causas potencialmente reversíveis de ansiedade e agitação, como dor, prurido, retenção urinária, constipação, insônia, irritabilidade e efeitos adversos de medicamentos, especialmente corticosteroides^22^. É fundamental realizar o diagnóstico diferencial, uma vez que condições como transtorno de pânico, delirium hiperativo, acatisia e transtorno de ansiedade social podem simular ansiedade, enquanto luto, tristeza, delirium hipoativo, demências e distúrbios metabólicos podem ser confundidos com depressão^53,54^.

Recomenda-se que todos os pacientes com DRC em MCA sejam sistematicamente avaliados para depressão e ansiedade, utilizando critérios diagnósticos estabelecidos no *Diagnostic and Statistical Manual of Mental Disorders* — DSM-5-TR e instrumentos de avaliação validados, como o PHQ-9 (para depressão) e o GAD-7 (para ansiedade), como ferramentas auxiliares para rastreamento, quantificação da gravidade dos sintomas e monitoramento da resposta ao tratamento^55^.

O manejo dos transtornos depressivos e ansiosos deve ser multidimensional e multiprofissional, com foco na qualidade de vida e na preservação da dignidade. Intervenções psicossociais, particularmente a TCC, apresentam evidência consistente de redução dos sintomas depressivos e ansiosos, além da melhora da qualidade de vida, sendo recomendadas por seu baixo risco^56,57^. Psicoterapias de base existencial, como a terapia da dignidade, a logoterapia e a revisão terapêutica da vida, são especialmente úteis no manejo da ansiedade, auxiliando na construção de significado e no alinhamento de valores e prioridades^58^.

Não há evidências robustas que indiquem uma classe específica de antidepressivos como primeira linha na DRC em MCA^59^. A escolha deve ser individualizada, considerando comorbidades, efeitos adversos, interações medicamentosas, função renal e preferências do paciente. Os inibidores seletivos da recaptação da serotonina (ISRS) podem ser utilizados com cautela, devido às alterações na farmacocinética na DRC e aos riscos potenciais, como sangramentos, eventos cardiovasculares e fraturas^59,60^. Além da sertralina, incluem-se citalopram, escitalopram, fluoxetina e paroxetina. Outras opções são os inibidores da recaptação de serotonina e noradrenalina (venlafaxina, duloxetina e mirtazapina), antidepressivos atípicos (bupropiona e trazodona) e os tricíclicos (amitriptilina), com monitorização rigorosa^60^.

No manejo da ansiedade, os benzodiazepínicos de curta ou intermediária duração (lorazepam, clonazepam ou diazepam) podem ser utilizados com cautela devido ao risco de sedação excessiva, delirium e quedas. Recomenda-se titulação lenta, doses iniciais reduzidas e reavaliação frequente (Quadro 2)^22,61^

**Quadro 2.** Antidepressivos e ansiolíticos na DRC.

Nome	Remoção na diálise	Dose (mg/dia)	Comentários
**Antidepressivos**			
Sertralina	Não	Inicial: 25 1x/d DRC: 50–200	1a linha. Geralmente segura.
Fluoxetina	Não	20–60	Sem ajuste na DRC.
Amitriptilina	Não	Inicial: 10 à noite e ajuste até 150	Não é de 1a linha. Tricíclico. Utilize com cautela. Uso limitado.
Escitalopram	Não	10–20	Sem ajuste na DRC.
Citalopram	Não	Inicial: 10 à noite e ajuste até 40	Risco de prolongamento do QT (ECG). Utilize com cautela.
Nortriptilina	Não	Inicial: 10 à noite e ajuste até 75–100	Não é de 1a linha. Utilize com cautela. Uso limitado. Tricíclico.
Trazodona	Não	Inicial: 25–50 e ajuste até 300 Para insônia: máximo 150	Não é de 1a linha. Utilize com cautela. Antidepressivo atípico.
Duloxetina	Não	Iniciar: ≤ 30 à noite até 120 Sem ajuste até TFG > 30 mL/min /1,73 m2	Não é de 1a linha. Utilize com cautela. Não recomendada para DRC V e diálise.
Mirtazapina	Não	Iniciar: 15 à noite Até DRC IV: 15–45 DRC V e diálise: corrigir em 50%	Não é de 1a linha. Utilize com cautela. Meia-vida longa, com possível demora no aparecimento de efeitos colaterais.
Venlafaxina	Não	Iniciar: 37,5 à noite até 112,5 em doses fracionadas Até DRC IV: reduzir a dose em 25%–50% DRC V e diálise: corrigir em 50%	Não é de 1a linha. Utilize com cautela. Em HD: administrar a dose após a sessão.
**Ansiedade e Insônia**			
Lorazepam	Não	Ansiedade: 2–3 VO ou SL até 2 mg 6/6h Insônia: iniciar 1–2 VO ou SL	Geralmente seguro. Doses menores podem ser suficientes.
Zolpidem	Não	Iniciar: 5 à noite, podendo chegar até 10	Geralmente seguro. Doses menores podem ser suficientes.
Zopiclona	Não	Iniciar: 3,75 à noite, podendo chegar até 7,5	Geralmente segura. Doses menores podem ser suficientes.
Clonazepam	Não	Iniciar: 0,25–0,50 e manter 0,50–2 6/6h	Utilize com cautela. Doses menores podem ser suficientes.
Diazepam	Não	5–10	Utilize com cautela e evite, se possível. Doses menores podem ser suficientes.
Bupropiona	Não	150–450	Utilize com cautela. Doses menores e/ou menor frequência podem ser necessárias.

Fonte: Adaptada de *Palliative Care Formulary*, 8ª edição^61^.

Estratégias não farmacológicas complementares, como exercício físico regular e acupuntura, demonstraram benefícios na redução dos sintomas depressivos e ansiosos em pacientes com DRC^62,63^. Técnicas de relaxamento, meditação, musicoterapia, espiritualidade, religiosidade e programas de promoção da resiliência também contribuem para a melhora do bem-estar psicológico e da qualidade de vida^64,65,66^.

O fortalecimento do suporte social e a atuação integrada de equipes multiprofissionais – envolvendo nefrologistas, psiquiatras, psicólogos e demais profissionais – são essenciais para uma abordagem integral, humanizada e segura, minimizando riscos, polifarmácia e interações medicamentosas^49^.

## CONSTIPAÇÃO

A constipação intestinal é uma queixa frequente em pacientes com DRC, embora a prevalência exata permaneça incerta devido à heterogeneidade dos critérios diagnósticos utilizados e à escassez de estudos robustos. Os critérios de Roma IV são amplamente utilizados e definem a constipação com base na presença de pelo menos dois dos seis sintomas específicos (Quadro 3)^67,68^.

**Quadro 3:** Critérios de Roma IV para constipação. Adaptado de Lacy e Patel^68^.

Presença de 2 ou mais sintomas: Esforço ao evacuar em > 25% das evacuações; Fezes endurecidas ou fragmentadas em > 25% das evacuações; Sensação de esvaziamento incompleto em > 25% das evacuações; Sensação de bloqueio ou obstrução anorretal em > 25% das evacuações; Uso de manobras manuais para facilitar a evacuação em > 25% das evacuações; Menos de 3 evacuações por semana.

Na DRC, a constipação associa-se à piora da qualidade de vida, ao aumento de custos em saúde e a complicações clínicas relevantes, incluindo acúmulo de toxinas urêmicas e hipercalemia. Sua etiologia é multifatorial, envolvendo sedentarismo, ingestão inadequada de fibras, uso crônico de medicamentos, neuropatia diabética, hipotireoidismo, neoplasias e distúrbios eletrolíticos, especialmente hipercalcemia^67^.

O manejo deve priorizar a identificação e a correção de causas reversíveis, com uma abordagem inicial não farmacológica. As medidas de primeira linha incluem incentivo à atividade física, adequação da ingestão hídrica e alimentar e orientações comportamentais, como responder ao reflexo evacuatório e adotar postura adequada durante a evacuação.

Do ponto de vista nutricional, o consumo de cereais integrais, frutas, vegetais e leguminosas favorece o trânsito intestinal pelo aporte de fibras e compostos probióticos. Contudo, esses alimentos também são fontes relevantes de

potássio e fósforo, exigindo avaliação nutricional individualizada em pacientes com DRC^69^.

Estratégias para aumentar a ingestão de fibras com menor risco de hipercalemia incluem a seleção de frutas e vegetais crus com menor teor de potássio e o pré-cozimento em água de vegetais e leguminosas. O feijão, importante fonte de fibras na dieta brasileira, pode ser utilizado, quando preparado adequadamente, para reduzir o teor de potássio. Ressalta-se ainda que o fósforo de origem vegetal apresenta menor biodisponibilidade, representando menor risco de hiperfosfatemia.

A suplementação de fibras (*psyllium*, goma-guar e farelo de trigo) e o uso de azeite de oliva como agente emoliente podem ser considerados, com introdução gradual e monitorização da resposta clínica^69^.

Na ausência de resposta às medidas não farmacológicas, pode ser necessário tratamento farmacológico com laxantes osmóticos, estimulantes entéricos e agentes pró-cinéticos. Em casos refratários, recomenda-se a investigação de disfunções do assoalho pélvico, que podem perpetuar a constipação crônica^68,70^.

## DISFUNÇÃO SEXUAL (DSEX)

A DSex é altamente prevalente em pacientes com DRC e associa-se à ansiedade, à depressão e à piora da qualidade de vida^9^. Nos homens, as principais manifestações incluem a disfunção erétil (DE), distúrbios da ejaculação, desejo sexual hipoativo e redução da libido, enquanto nas mulheres predominam dispareunia, diminuição da lubrificação vaginal, desejo sexual reduzido e anorgasmia^71,72^.

Estima-se que a DE acometa cerca de 71% dos homens com DRC, alcançando 68–92% entre os pacientes em hemodiálise. Alterações ejaculatórias e anorgasmia são relatadas por aproximadamente 59% dos homens em TRS^73,74^. Entre as mulheres, dados limitados sugerem prevalência de DSex de até 84% em paciente em TRS^75^.

A DSex na DRC é multifatorial, envolvendo componentes físicos, hormonais, psicológicos e sociais. Fadiga, dor crônica e elevada carga de comorbidades, incluindo diabetes mellitus, hipertensão arterial, obesidade, neuropatias, doenças cardiovasculares e vasculopatias periféricas, contribuem significativamente para sua ocorrência^73^.

As alterações endócrinas, particularmente a disfunção do eixo hipotálamo-hipófise-gonadal, tornam-se mais pronunciadas com a progressão da DRC^76^. Medicamentos frequentemente utilizados, como betabloqueadores, diuréticos, simpatolíticos, corticosteroides, inibidores da enzima conversora de angiotensina e bloqueadores dos receptores de angiotensina II, também podem agravar a DSex^77^.

Nas mulheres, o hipoestrogenismo é fator central, associado a menopausa precoce, redução do fluxo sanguíneo genital, diminuição da lubrificação vaginal e dispareunia^73,78^. A síndrome geniturinária da menopausa é comum e inclui sintomas genitais (atrofia, ressecamento, ardor, dispareunia) e urinários (disúria, urgência miccional, incontinência urinária e infecções recorrentes)^79^.

A DE na DRC avançada resulta da combinação de alterações hormonais, disfunção endotelial, neuropatia autonômica e neuropatia urêmica, com prejuízo da resposta neurovascular peniana^71,73,80^. Hiperprolactinemia, redução da testosterona e alterações no eixo hipotálamo-hipófise-gonadal são achados frequentes^80^.

A avaliação deve ser ativa e empática, uma vez que os sintomas são raramente referidos espontaneamente. O *International Index of Erectile Function* (IIEF) é um instrumento validado de referência, sendo o IIEF-5 útil para triagem e monitoramento da gravidade da DE, embora não identifique sua etiologia (Quadro 4)^81^.

**Quadro 4:** Índice Internacional de Função Erétil (IIEF) 5^81^.

**Nas últimas 4 semanas:**	
**Item 1**	**Com que frequência você conseguiu uma ereção durante a atividade sexual?**
Resposta	0. Não pude ter relações sexuais 1. Quase nunca ou nunca 2. Raramente (pouquíssimas vezes, considerando todas que já aconteceram) 3. Às vezes (mais ou menos na metade das vezes) 4. Na maior parte das vezes 5. Quase todas as vezes ou todas as vezes
**Item 2**	**Quando você teve ereções com estimulação sexual, com que frequência suas ereções foram duras o suficiente para penetração?**
Resposta	0. Não pude ter relações sexuais 1. Quase nunca ou nunca 2. Raramente (pouquíssimas vezes, considerando todas que já aconteceram) 3. Às vezes (mais ou menos na metade das vezes) 4. Na maior parte das vezes 5. Quase todas as vezes ou todas as vezes
**Item 3**	**Quando você tentou uma relação sexual, com que frequência você conseguiu penetrar em sua parceira?**
Resposta	0. Não pude ter relações sexuais 1. Quase nunca ou nunca 2. Raramente (pouquíssimas vezes, considerando todas que já aconteceram) 3. Às vezes (mais ou menos na metade das vezes) 4. Na maior parte das vezes 5. Quase todas as vezes ou todas
**Item 4**	**Durante a relação sexual, com que frequência você conseguiu manter a ereção depois de ter penetrado em sua parceira?**
Resposta	0. Não pude ter relações sexuais 1. Quase nunca ou nunca 2. Raramente (pouquíssimas vezes, considerando todas que já aconteceram) 3. Às vezes (mais ou menos na metade das vezes) 4. Na maior parte das vezes 5. Quase todas as vezes ou todas
**Item 5**	**Durante a relação sexual, quão difícil foi manter sua ereção até o fim da relação sexual?**
Resposta	0. Não pude ter relações sexuais 1. Extremamente difícil 2. Muito difícil 3. Difícil 4. Um pouco difícil 5.Não foi difícil

O manejo deve ser individualizado e incluir intervenções psicossociais e mudanças no estilo de vida. O controle otimizado de comorbidades, a correção da anemia, dos distúrbios do metabolismo mineral e das alterações hormonais podem resultar em melhora da função sexual. Recomenda-se cessação do tabagismo, atividade física regular e manejo do estresse.

Na DE, o tratamento de primeira linha consiste nos inibidores da fosfodiesterase tipo 5 (iPDE-5) (sildenafila, tadalafila, vardenafila, lodenafila), utilizados sob demanda ou, no caso da tadalafila, em regime diário. Em pacientes com DRC, recomenda-se iniciar com doses reduzidas, especialmente na presença de alfa-bloqueadores ou de medicamentos que inibem o CYP3A4. A principal contraindicação é o uso concomitante de nitratos^82^.

Na ausência de resposta, devem-se revisar aspectos relacionados ao uso adequado da medicação e avaliar hipogonadismo, cuja correção pode melhorar a resposta terapêutica. Em casos refratários, podem ser considerados o tratamento intracavernoso ou o implante de prótese peniana. Parte dos pacientes apresenta melhora após o transplante renal.

Pacientes com DE moderada a grave e refratária, hipogonadismo confirmado, deformidades penianas ou suspeita de etiologia não relacionada à DRC devem ser encaminhados ao especialista.

O manejo da dispareunia inclui lubrificantes e hidratantes vaginais e, na atrofia vaginal, estrogênio tópico, quando não houver contraindicações^83^. A fisioterapia do assoalho pélvico e a TCC são estratégias eficazes no tratamento da dor e dos fatores psicossociais associados^83,84^.

## CONCLUSÃO

A DRC apresenta elevada carga sintomática, especialmente em pacientes idosos, com múltiplas comorbidades e comprometimento funcional. Nesse contexto, o MCA constitui alternativa válida à TRS. A avaliação sistemática e proativa dos sintomas, associada a uma abordagem clínica individualizada, é fundamental para o controle adequado e para a promoção de cuidado centrado na pessoa. O MCA deve ser integrado precocemente à trajetória clínica, respeitando valores, preferências e prioridades do paciente.

## Data Availability

Nenhum dado novo foi gerado ou analisado neste estudo.
